# RACE/ETHNICITY AND THE MEASUREMENT OF COGNITION IN NSHAP: RECOMMENDATIONS FOR ROBUSTNESS

**DOI:** 10.1093/geroni/igad104.0232

**Published:** 2023-12-21

**Authors:** James Iveniuk, Haena Lee, Selena Zhong, Jocelyn Wilder, Gillian Marshall, Boyle Patricia, Lissette Piedra, Alicia Riley

**Affiliations:** NORC at the University of Chicago, Chicago, Illinois, United States; Sungkyunkwan University, Seoul, Republic of Korea; NORC, Chicago, Illinois, United States; NORC, Chicago, Illinois, United States; NORC at the University of Chicago, Chicago, Illinois, United States; Rush Alzheimer’s Disease Center, Chicago, Illinois, United States; University of Illinois at Urbana-Champaign, Urbana, Illinois, United States; UC Santa Cruz, Santa Cruz, California, United States

## Abstract

In this study, we interrogate measurement of cognition by race, in order to move towards a less-biased and more-inclusive set of measures for capturing cognitive change and decline in older adulthood. We use data from Round 2 (N=3377) and Round 3 (N=4777) of the National Social Life Health and Aging Project (NSHAP), and examine the study’s version of the Montreal Cognitive Assessment (MoCA). We employ exploratory factor analyses to explore configural invariance by racial/ethnic group (Non-Hispanic White, Non-Hispanic Black, Hispanic, All else), and then log-likelihood tests of scalar and metric invariance. Using modification indexes we identify items that seem robust to bias by race. We test the predictive validity of the full (18-item) and short (7-item) scales using self-reported dementia diagnosis, Instrumental Activities of Daily Living (IADLs), proxy reports of dementia death, and National Death Index (NDI) reports of dementia death. We found that 7 measures, out of the 18 used in NSHAP’s MoCA, formed a scale that was more robust to racial bias. The shortened form predicted consequential outcomes equally well, compared to NSHAP’s full MoCA. The short form was also highly correlated with the full form, and displayed lower test-retest correlation between Round 2 and 3. Although sophisticated structural equation modeling techniques could be useful for assuaging measurement invariance by race in NSHAP, the shortened form provides a quick way for researchers to carry out robustness checks – to see if the disparities and associations they document are ‘real’ or the product of artifactual bias.

